# Targeting the lateral neck of superior articular process and sub-mammillary fossa for lumbar medial branch radiofrequency ablation: A case series

**DOI:** 10.1016/j.inpm.2024.100533

**Published:** 2024-11-30

**Authors:** John Tran, Nicole Billias, Taylor Burnham, Robert Burnham, Eldon Loh

**Affiliations:** a Department of Physical Medicine and Rehabilitation, Parkwood Institute, London, Canada; b Division of Anatomy, Department of Surgery, University of Toronto, Toronto, Canada; c Vivo Cura Health, Calgary, Alberta, Canada

## Abstract

**Introduction:**

Lumbar medial branch (MB) radiofrequency ablation (RFA) is a common image-guided procedure to treat facetogenic low back pain. Recent anatomical literature has proposed a two-lesion RFA approach targeting the posterior portion of the lateral neck of superior articular process (SAP) and the superior aspect of the sub-mammillary fossa. The objectives of this report were to: 1) describe the novel lumbar MB RFA technique, 2) discuss the relevant anatomy, and 3) report pain relief outcomes in four patients who gave informed consent to be treated with the novel two-landmark lumbar MB RFA technique.

**Methods:**

Four patients were treated with the novel two-landmark lumbar MB RFA technique targeting the posterior half of the lateral neck of SAP and superior aspect of the sub-mammillary fossa. The quality and duration of pain relief following the treatment are described in this report.

**Results:**

All 4 patients, who received the novel technique, self-reported quality of pain relief of ≥80 %. One patient, who self-reported 100 % pain relief, elected not to have repeat RFA treatment at their 15-month follow-up appointment. All 4 patients reported pain relief duration ≥12 months and stated the quality of pain relief following procedure was “excellent” or the “best result” they experienced.

**Conclusions:**

This case series reports early evidence of the effectiveness of the two-landmark lumbar MB RFA technique. The novel approach shows promise in a limited number of patient cases and warrants further investigation.

## 1 Introduction

**Fig. 1:**
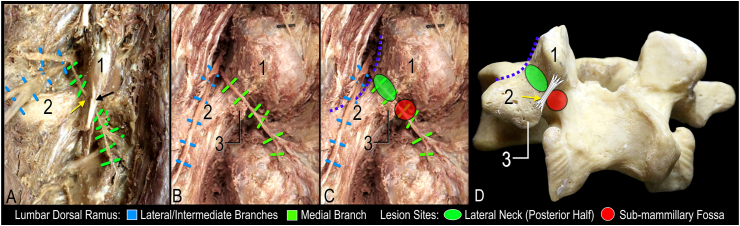
**Dissection images and lumbar vertebra illustrating proposed lesions in relation to the medial branch and bony anatomy.** A. Dissection showing course of medial branch coursing under mamillo-accessory ligament (yellow arrow) and tendon of longissimus thoracis pars lumborum (black arrow). B. Course of medial branch along lateral neck and through the sub-mammillary fossa; note the mamillo-accessory ligament and tendon of longissimus thoracis pars lumborum have been removed. C. Overlay of lesions at the two proposed target sites relative to the course of the medial branch in B. D. Bony lumbar vertebra with illustration of anatomical relationship of lesion, mamillo-accessory ligament and bony landmarks. Purple dotted line indicates contour of the junction between neck of the superior articular process and base of transverse process; 1, mammillary process; 2, transverse process; 3, accessory process. (For interpretation of the references to colour in this figure legend, the reader is referred to the Web version of this article.)

Lumbar medial branch (MB) radiofrequency ablation (RFA) is a common image-guided procedure to treat facetogenic low back pain [1]. Detailed anatomical knowledge is essential to optimize lumbar MB RFA. Recent anatomical studies have applied modern technologies to perform 3D spatial analysis of the relationship of the MB to relevant bony landmarks of the lumbar vertebra [2,3]. Utilizing a nerve proximity mapping methodology, the MB course was visualized on the lateral neck of the superior articular process (SAP). Two anatomical landmarks were reported to be consistent targets to potentially capture the nerve [3]. The first target site was proximal to the mamillo-accessory notch (at the posterior portion of the lateral neck of SAP), and the second was distal to the notch (inferior to the mammillary process) (Fig. 1). Review of current anatomy textbooks does not describe the second target site [4,5]. The term sub-mammillary fossa will reference the anatomical landmark inferior to the mammillary process [3].

Based on the consistent course of the MB, as described in previous anatomical literature [6,7] and more recent 3D studies [2,3], a two-lesion approach was proposed targeting the posterior portion of the lateral neck of SAP and the superior aspect of the sub-mammillary fossa [3]. Currently, no technical description or clinical data exists for this anatomy-informed approach. Therefore, the objectives of this report were to: 1) describe the novel two-landmark lumbar MB RFA technique, 2) discuss the relevant anatomy, and 3) report pain relief outcomes in four patients who gave informed consent to be treated with the novel two-landmark lumbar MB RFA technique.

## 2 Case reports

Four patients were treated with the novel two-landmark RFA technique targeting the posterior half of the lateral neck of SAP and superior aspect of the sub-mammillary fossa. The pain relief outcomes following the treatment are described in this report. Each patient gave verbal informed consent to the novel RFA technique after explaining the anatomical basis. The senior author (EL) performed the procedures at St. Joseph's Health Care London (SJHC) Pain Clinic, a hospital-based tertiary pain clinic in London, Ontario, Canada. All patients received L3–L5 MB denervation between April 2023 and July 2023. The patients, following medial branch block (MBB), demonstrated ≥50 % relief for at least the duration of the local anesthetic prior to their initial RFA. MBB was not reported before repeat RFA. At three months post-procedure, patients had initial clinic follow-up to determine pain relief outcomes anecdotally. As part of routine practice, pain was assessed on a numeric rating scale (0–10, with 0 being no pain and 10 being worse pain imaginable). Patients were also asked if they had any complications or side effects from the procedure and were provided with contact information for the clinic and encouraged to call the clinic if there were any post-procedural concerns. When patients were scheduled to return for repeat ablation at 9–15 months post-procedure, pain relief outcomes, including duration, were again documented. This case series consisting of reported outcomes from a very small number of patients/cases (n = 4) from a single physician's practice was exempted from the Western University Health Sciences Research Ethics Board (HSREB).

### 2.1 Procedural technique

**Fig. 2:**
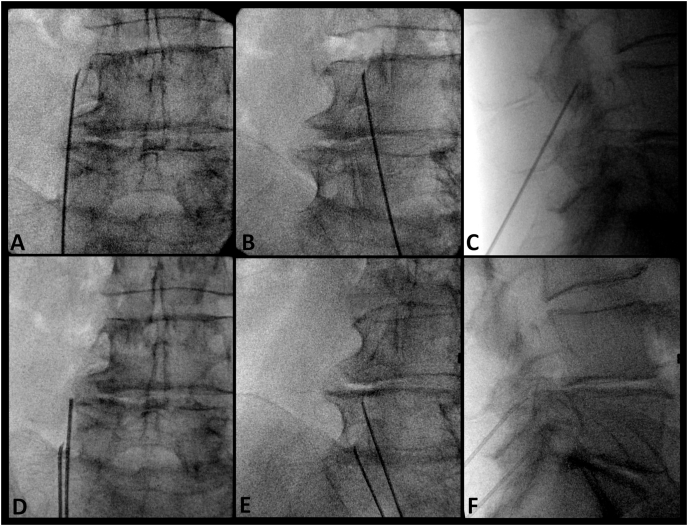
**Fluoroscopic images of cannula placement technique targeting posterior half of lateral neck of superior articular process.** Conventional cannula placement targeting L3 medial branch: A. Postero-anterior view. B. Oblique view. C. Lateral view. Conventional cannula placement targeting L4 and L5 medial branches: D. Postero-anterior view. B. Oblique view. C. Lateral view.

**Fig. 3:**
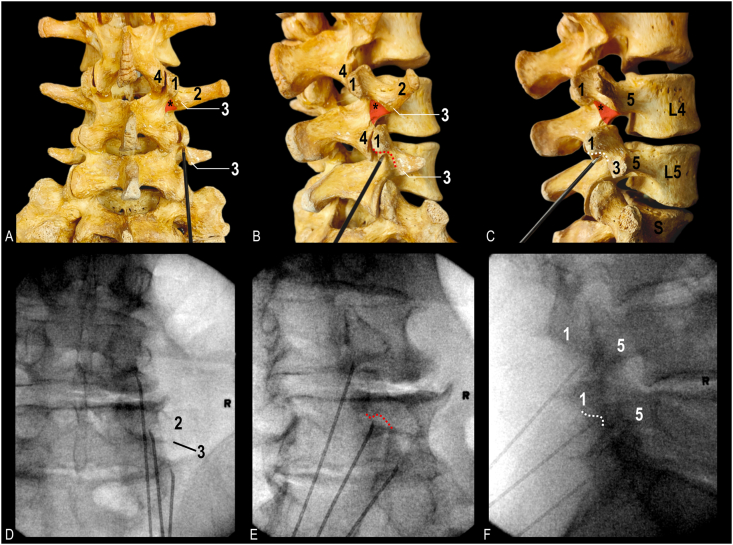
**Bony anatomy and fluoroscopic images of cannula placement technique targeting sub-mammillary fossa.** A. Posterior view of bony lumbosacral spine with cannula position at sub-mammillary fossa of fifth lumbar vertebra. B. Oblique view. C. Lateral view. D. Radiographic image with cannula targeting the sub-mammillary fossa at the L3 and L4 medial branch level; note cannula targeting L5 medial branch is positioned along the lateral neck. Postero-anterior view. E. Oblique view. F. Lateral view with all 3 cannulae positioned at sub-mammillary fossa. Asterisk indicates second target at superior aspect of sub-mammillary fossa (red triangle); red dotted curve, contour of mamillo-accessory notch seen in oblique view; white dotted curve, contour of mamillo-accessory notch seen in lateral view; 1, mammillary process; 2, transverse process; 3, accessory process; 4, inferior articular process; 5, pedicle; S, sacrum. (For interpretation of the references to colour in this figure legend, the reader is referred to the Web version of this article.)

As previously published, a parasagittal cannula approach was first performed to target the posterior half of the lateral neck of SAP [8]. Using fluoroscopy, a lateral view was obtained. A metal pointer was used to identify two landmarks: 1) the mamillo-accessory notch and 2) a point on the anterior border of the SAP at the level of the superior vertebral endplate. An imaginary line connecting the two landmarks was used to estimate the trajectory to guide a cannula parallel along the MB. After identifying the approximate cannula trajectory, a mark was made on the patient's exposed back to estimate how far distally the cannula needed to be inserted to achieve placement along the estimated trajectory of the MB. Next, a postero-anterior (PA) view was obtained. The PA view was declined caudally until the SAP/transverse process was no longer easily visible (usually up to 30°); this was to bring the target area closer to the mark on the skin made in the prior step. Using the neck of the SAP as a target, a second mark to achieve a parasagittal placement was made at the level of the distal entry site marked previously. This second skin mark represented the cannula entry point. The cannula was then inserted and guided to the target area at the lateral neck of SAP. After placing the cannula, oblique, lateral, and PA views were obtained to confirm correct cannula placement (Fig. 2). The senior author's practice is to unilaterally place up to three cannulae simultaneously (if possible) to ablate the L3, L4, and L5 MBs together in one cycle. Before ablation, motor stimulation was performed to confirm safe placement. The procedure used a 10-cm or 15-cm 18-gauge RFA cannula with a curved, 10-mm exposed tip (Diros Technologies, Markham, Canada) to reach the target area due to the degree of caudal angulation needed to achieve parallel trajectory with MB. A small amount of local anesthetic was injected at each level (1 % lidocaine), and the ablation was carried out for 120s at 80 °C after a 30s ramp time (150s total). Following ablation using the parasagittal approach (targeting the posterior half of the lateral neck of SAP), the cannula was retracted slightly. A PA view (with vertebral endplate squared) was obtained to identify the sub-mammillary fossa located just below the inferior margin of the mammillary process. The cannula was then redirected toward the sub-mammillary fossa and advanced until bone contact (Fig. 3). A 30-degree ipsilateral oblique view was obtained to confirm placement and ensure the cannula tip was located just medial and inferior to the mamillo-accessory notch (Fig. 3E). A lateral view was then obtained to substantiate that the cannula was in contact with the periosteum, inferior to the mammillary process (i.e., the superior aspect of the sub-mammillary fossa). Following repositioning, all the cannulae and injection of a small amount of 1 % lidocaine for comfort, motor stimulation was performed to confirm safe placement. The ablation was carried out for 120s at 80 °C after a 30 s ramp time (150s total).

### 2.2 Patient outcomes

Four patients received L3-L5 MB denervation using a parasagittal and sub-mammillary technique. Patients reported no serious adverse effects following the RFA treatment using the two-landmark approach. The level of discomfort during the procedure was noted to be similar to previous visits. A description of each patient case is provided below. Patient characteristics and pain relief outcomes are summarized in Table 1.

**Table 1:** Patient characteristics and pain relief outcomes following L3-L5 medial branch RFA using parasagittal and sub-mammillary fossa technique.

Case	Age/Sex	Total duration (months) between RFA treatments	Quality (%) and duration (months) of pain relief
1	70F	14 months^a^	Partial relief (90 %) for 13 months
			Partial relief (75 %) for 1 month
2	54M	12 months^a^	Partial relief (90 %) for 11 months
			Partial relief (10–20 %) for 1 month
3	78F	15 months^b^	Complete relief (100 %) for 15 months
4	75F	17 months^a^	Partial relief (80 %) for 16 months
			Partial relief (25 %) for 1 month

a Pain did not return to baseline, but patient elected to have repeat RFA procedure.

b No recurrence of symptoms patient elected not to have repeat RFA treatment RFA, radiofrequency ablation.

### 2.3 Case 1

A 70-year-old female suffering from low back pain with right radicular pain since 2016. While lumbar epidural injection provided relief of lower extremity symptoms, the patient had ongoing low back pain radiating to both hips. The patient received bilateral L3-L5 MB RFA using the parasagittal and sub-mammillary technique. Fourteen months following the procedure, the patient elected to have repeat RFA treatment while self-reporting “excellent” (90 %) pain relief for 13 months and ongoing partial (75 %) pain relief.

### 2.4 Case 2

A 54-year-old male patient suffering from low back pain following a motor vehicle collision received bilateral L3-L5 MB denervation. Following the parasagittal and sub-mammillary technique, the patient self-reported excellent (90 %) pain relief for 11 months. At 12 months, the patient had ongoing partial (10–20 %) pain relief and elected to have repeat RFA treatment. The patient stated this was the “best result to date” from RF ablation in terms of quality of pain relief.

### 2.5 Case 3

A female 78-year-old patient received L3-L5 MB denervation on the right side using the parasagittal and sub-mammillary technique. At 15 months following RFA treatment the patient self-reported complete (100 %) pain relief that is ongoing with no recurrence of symptoms. The patient stated this was the “best result ever” from any previous RFA and elected to delay repeat treatment.

### 2.6 Case 4

A 75-year-old female was treated with bilateral L3-L5 MB denervation using the parasagittal and sub-mammillary technique. At the 17 months post-procedure appointment, the patient self-reported partial pain relief (80 %) for 16 months and 1 month of ongoing relief at 25 %. The patient stated this was the “best result of any ablation” previously received.

## 3 Discussion

Previous anatomical research proposed a two-landmark lumbar MB RFA technique to extend the length of the nerve target being captured [3]. In the current report, early clinical outcomes in 4 patients who consented to the anatomy-informed technique were reported. Additionally, a description of the technique is provided with corresponding anatomy and fluoroscopic images, providing spine-pain interventionalists with a resource to incorporate this technique into their repertoire and further assess the clinical effectiveness of the approach.

### 3.1 Relevant anatomy

The MB has been consistently described to course along the lateral neck of the SAP, passing through the mamillo-accessory notch deep to the mamillo-accessory ligament [2,[6], [7], [8], [9], [10]]. The traditional approach of targeting the nerve along the lateral neck of the SAP was based on the rationale that the MAL may insulate the MB from coagulation [10]. While this approach only captures the MB proximal to the MAL, the current two-landmark lumbar MB RFA technique also targets the nerve distal to the MAL (Fig. 1). Specifically, the two-landmark lumbar MB RFA technique targets the posterior half of the lateral neck of the SAP [7], proximal to the MAL, and the superior aspect of the sub-mammillary fossa, distal and medial to the MAL (Fig. 1C). This cannula placement relative to the MB may account for the promising pain relief outcomes reported by patients in the current case series. However, anatomical variations and degenerative changes do exist. Therefore, variable outcomes are to be expected.

### 3.2 Quality of pain relief and duration

In the current case series, all 4 patients who received the novel two-landmark lumbar MB RFA technique self-reported quality of pain relief of ≥80 %. One patient, that self reported 100 % pain relief (case 3), elected not to have repeat RFA treatment at their 15-month follow-up appointment. Interestingly, all four patients stated the quality of pain relief following the novel two-landmark lumbar MB RFA technique was “excellent” or the “best result” they experienced. This may be attributed to the number of axons coagulated during the RFA procedure. With the addition of the second lesion (at the sub-mammillary fossa) any axons not coagulated by the first lesion (at the lateral neck of the SAP) may be potentially captured at the second site. Further, if there are any anatomical variations at the lateral neck of the SAP (osteophyte impeding proper placement of the cannula) or technical factors that result in a suboptimal cannula placement (e.g. insufficient caudal angulation), the second placement may ensure that the MB is captured if it is missed during initial placement at the lateral neck of the SAP, increasing the chances of procedural success.

The duration of pain relief following the RFA procedure is also promising. In all four patients who received the novel approach, pain relief duration was ≥12 months. This outcome is comparable with the reported duration of other MB denervation approaches [8,[11], [12], [13]]. It is anticipated that optimization of pain relief quality and duration using the novel two-landmark lumbar MB RFA approach will require further anatomical and clinical investigation. Future anatomical studies are necessary to validate the fluoroscopic features used to target the sub-mammillary landmark, which may lead to more accurate cannula placement and could maximize the length of MB coagulated. Clinical studies are necessary to assess the safety and effectiveness of this novel technique while also determining RFA lesion configurations that optimize pain relief outcomes.

### 3.3 Procedural technique and considerations

In the current case series, a parasagittal approach was used first to target the posterior half of the lateral neck of SAP. This approach was informed by previous anatomical and clinical literature [8,14]. However, anatomical variations are expected, which may impact the ability of one single approach to provide consistent benefits for all patients. Therefore, spine pain interventionalists should develop a repertoire of different techniques (i.e., parasagittal, traditional, perpendicular) to provide a patient-specific approach for coagulating the greatest length of the MB along the lateral neck of SAP. Future studies are necessary to identify fluoroscopic features to determine patient-specific approaches to target the lateral neck of the SAP.

Additionally, while the approach of performing two RFA burns has been previously described, the previous technique involved repositioning the cannula at the same landmark to optimize the capture of the MB [15]. This accommodated variations of the MB as it courses along the lateral neck of SAP and may achieved more consistent patient outcomes. In contrast, the two-landmark lumbar MB RFA technique described in the current report targets two unique landmarks where the MB has been found consistently [3]. If a bony obstruction or some other technical factor impedes the initial placement, a second ablation in the same area may not overcome this barrier. However, targeting a second area that is distinct from the first target may increase overall chances of success. Future clinical investigation is required to assess this postulation.

In this case series, the second ablation at the sub-mammillary fossa was completed using a perpendicular technique. Therefore, the area captured during the ablation would be limited by the configuration of the lesion, as a conventional electrode and cannula were used. It is possible that other cannula types (e.g. multi-tined/expanded lesions) would optimize capture at the second target site. However, rather than utilize a separate large lesion electrode, it was most practical to slightly retract and redirect the cannula medial towards the sub-mammillary fossa, particularly given the proximity of the two target sites. Future studies should evaluate using multi-tined/expanded lesion electrodes at the second target (together with a parallel approach at the first target). Evaluation of isolated perpendicular lesions at the sub-mammillary fossa target (either with conventional or multi-tined/expanded lesion electrodes) and perpendicular lesions with multi-tined/expanded lesions at both sites would be important for future studies to address.

From a risk perspective, the MB has separated from the lateral and intermediate branches of the lumbar dorsal ramus at the sub-mammillary fossa. Therefore, the risk of iatrogenic injury with larger/expanded lesions is relatively low as compared to targeting the lateral neck of SAP. Although no complications were observed in the current report, further cadaveric research is necessary to elucidate the anatomical structures close to the sub-mammillary fossa to assess safety and potential risks when using expanded lesions.

### 3.4 Limitations

The findings of this case series publication are limited by bias, overinterpretation, and inability to generalize to the greater population [16]. However, it provides the means to generate new research questions and future clinical studies to improve patient care. The present report provides preliminary evidence of the effectiveness of an anatomy-informed two landmark lumbar MB RFA technique. The reported patient outcomes support the need for a clinical pilot study to assess safety and effectiveness. The outcome of the pilot study may warrant further investigation in the form of a randomized clinical trial.

## 4 Conclusions

In the current case series, early clinical evidence of the effectiveness of the two landmark lumbar MB RFA technique was reported. Patients reported no serious adverse effects. The novel approach, informed by robust anatomical evidence, shows promise in a limited number of patient cases and warrants further investigation. This study provides technical description, images of cannula placement and pilot outcomes of this innovative technique. Future anatomical and clinical research is necessary to confirm the safety and effectiveness of the two landmark lumbar MB RFA technique.

## Declaration of competing interest

The authors declare that they have no known competing financial interests or personal relationships that could have appeared to influence the work reported in this paper.
